# Restricting use of adenovirus vector-based COVID vaccines could endanger public and global health

**DOI:** 10.3389/fimmu.2022.985382

**Published:** 2022-08-25

**Authors:** Hildegund C. J. Ertl, Sue L. Currie, Andrew D. Luber

**Affiliations:** ^1^ The Wistar Institute, Philadelphia, PA, United States; ^2^ Virion Therapeutics, LLC, Newark, DE, United States

**Keywords:** VITT, COVID, adenoviral (Ad) vector, vaccines, global public health, thrombosis with thrombocytopenia syndrome (TTS), myocarditis, benefit-risk strategies

On May 5, 2022, the United States (US) Food & Drug Administration (FDA) severely restricted the use of the Johnson & Johnson’s (J&J) adenovirus (Ad) serotype 26 (HAdV-26) COVID-19 vaccine to individuals unable or unwilling to receive one of the mRNA vaccines. The FDA decision was based on the rare, but potentially fatal, thrombosis with thrombocytopenia syndrome (TTS) that has been linked to Ad vector-based COVID vaccines. As a result, the US population is now largely dependent on mRNA COVID vaccines from Pfizer or Moderna. While concerning, the risk of TTS is extremely low and the restriction of Ad vector-based vaccines is premature, as our understanding of COVID - and COVID vaccine-induced immune responses and related risks and benefits - continues to evolve.

Ad vector-induced TTS generally occurs within 21 days following COVID-19 vaccination; to date this serious adverse event (SAE) has not been observed following Ad vector-based vaccinations for other infectious diseases. In the US, 60 TTS cases and 9 deaths have been reported out of 194 million vaccine recipients (1). The incidence of TTS varies by region; Nordic European countries, which are using the Astra Zeneca (AZ) COVID-19 vaccine, reported 17.6 cases of TTS per million doses, which is above the rate of 5.6 to 10.6 vaccine-independent events per million in the general population. However, only 0.2 AZ vaccine-linked cases per million doses were observed in Asia or Brazil (2). Potential explanations for these findings include regional differences in SAE reporting or differences in the vaccine recipients’ health status or genetic make-up. TTS has not been observed after immunization with Sputnik V, an Ad vector-based COVID-19 vaccine from the Gamaleya Institute that uses HAdV-26 and HAdV-5 for priming and boosting, respectively - millions of doses have been given in over 70 countries, thereby making it highly unlikely that TTS was simply “overlooked”.

The precise etiology for vaccine-related TTS remains unknown. Patients with TTS develop thrombocytopenia and autoantibodies against platelet factor 4 (PF4); the latter promote blood clot formation (3). Some *in vitro* studies suggest that these antibodies are induced by complexes formed between the negatively charged PF4 and the positively charged Ad hexon, but the finding that the HAdV-26 and HAdV-5 vectors of Sputnik V are not causing TTS contradicts this theory. It is also difficult to reconcile why a replication-defective vaccine given into the muscle causes TTS while an Ad infection that can result in viremia - and even death - has not been linked to this disease. Alternative TTS triggers, such as contaminants or components of the formulation buffer have been associated with this event and should be further explored (4).

When weighing the risk-benefit ratio of any medical intervention, one must assess the alternative options which, in the US, for COVID-19, are mRNA vaccines, which cause anaphylaxis and myocarditis (especially in younger males) events of 2.5 - 11.5 and 21 – 107 per million vaccine recipients, respectively. While generally well tolerated, severe cases of mRNA- induced myocarditis requiring hospitalization and death have been reported (5–7). Additionally, some mRNA vaccine recipients experience rather unpleasant systemic reactions, and, as was shown for the Moderna vaccine, some develop antibodies against lipids of the vaccine formulation (8).

The pharmacodynamics of Ad vectors and mRNA vaccines differ – Ad vectors persist at low levels for several weeks, whereas mRNA is cleared rapidly; this in turn is likely to affect the duration and composition of the ensuing immune responses. For COVID-19, mRNA vaccines show high efficacy against disease following the initial 2 vaccine doses. Unfortunately, immune responses and protection taper off within 6 months, necessitating additional booster immunizations (9). However, protection from a second and third boost, given to vulnerable individuals, declines after 4 and 2 months, respectively, which is in part driven by new SARS-CoV-2 variants, but also by a rapid waning of vaccine-induced SARS-CoV-2-specific immune responses (10). Booster immunizations every 4-6 months (or sooner) to maintain protective immunity is too resource-intensive and costly to be feasible. Frequent boosts could potentially also have unwanted long-term effects on the immune system.

Immune responses upon Ad vector vaccination decay over time but less rapidly than those induced by mRNA vaccines. Ad vectors not only induce insert-specific antibody responses but also exceptionally potent CD8^+^ T cell responses (11). CD8^+^ T cell memory is long lived and CD8^+^ T cells, especially those that are still partially activated, can commence effector functions immediately unlike memory B cells that typically take a few days to differentiate into antibody-secreting plasma cells. Why is this important for COVID-19 vaccines? CD8^+^ T cells cannot prevent an infection, which none of the current vaccines achieves in any case, but by killing cells early after infection and before new viral progeny has been produced CD8^+^ T cells can block viral spread within a patient and thereby shorten the infection, lessen the severity of disease, and decrease transmission to others.

SARS-CoV-2 will continue to mutate, and evolving variants will at least in part be driven by their ability to evade neutralizing antibodies. Making new vaccines that express the spike protein from variants is technically feasible, but impractical, considering that variants such as omicron sweep the globe within weeks, whereas making enough vaccine for large-scale use takes months. It’s also not certain if variant-specific vaccine will indeed increase immune responses against the new strain and not simply boost antibodies that had been induced by the initial vaccine. Some of the CD8^+^ T cell epitopes may be lost upon mutations, but most will remain intact especially if COVID-19 vaccines start to incorporate additional internal viral proteins that tend to be more conserved than the spike protein, which is the only SARS-CoV-2 antigen that is expressed by current mRNA and Ad vector vaccines.

Ad vector-based vaccines remain a key strategy in our fight against COVID-19. Ad vectors induce more durable responses, they are less costly than RNA vaccines and they have a wider range of effective doses. In contrast to mRNA vaccines, Ad vectors do not require storage at sub-zero temperatures, but can be kept for days at ambient temperatures; this factor is especially important for resource-poor countries, which lack the capacity to maintain cold-chain conditions necessary for storage. Developing countries commonly look for US regulatory agencies for guidance and will likely be influenced by the FDA’s ruling on Ad vector-based COVID-19 vaccines. Clearly Ad vector vaccine regimens could be optimized. Simian Ad vectors could replace those based on common human serotypes to which humans have neutralizing antibodies that reduce the vectors’ immunogenicity. Use of the same vector twice in homologous prime boost regimens where the 1^st^ dose of the vaccine will induce Ad neutralizing antibodies, which again will reduce the immunogenicity of the boost, should be replaced with heterologous prime boost regimens using either two serologically distinct Ad vectors or combining an Ad vector with a different vaccine platform.

COVID-19 vaccine options remain limited and to restrict access to Ad vector vaccines, which have advantages over mRNA vaccines, is at this stage, not only counterproductive – but also could endanger public and global health.

## Author contributions

All authors listed have made a substantial, direct, and intellectual contribution to the work and approved it for publication.

## Conflict of interest

HE has the following disclosures: Co-founder of Virion Therapeutics, Advisor roles (board or consultancy) with: Freelance, Inc, Takeda, Biogen (board), Regenxbio, Ring Therapeutics (board), Canine Rabies Treatment Initiative (board). SC has the following disclosures: COO, Virion Therapeutics, Stock, Nektar Therapeutics. AL has the following disclosures: CEO, Virion Therapeutics.

## Publisher’s note

All claims expressed in this article are solely those of the authors and do not necessarily represent those of their affiliated organizations, or those of the publisher, the editors and the reviewers. Any product that may be evaluated in this article, or claim that may be made by its manufacturer, is not guaranteed or endorsed by the publisher.
